# Facial swelling in newborn

**DOI:** 10.1590/S1679-45082014AI2606

**Published:** 2014

**Authors:** Fabiana Fortunato, Irina Carvalheiro, Cristina Novais, Raquel Carreira, Nádia Brito

**Affiliations:** 1 Centro Hospitalar Oeste Norte, Caldas da Rainha, Portugal.

A 22-day-old male infant, the first child of non-consanguineous parents, who was delivered from a followed-up and normal pregnancy, and without alterations in analytical and echographic evaluation. He was born by cesarean section at 39 weeks, because the labor failed to progress, with Apgar score of 9 at 1 minute and of 10 at 5 minute, weighting 3,240g at birth without intercurrences in the neonatal period. The newborn was exclusively breastfed with adequate weight gain (with 22 days of life he weighed 3,650g).

**Figure 1:**
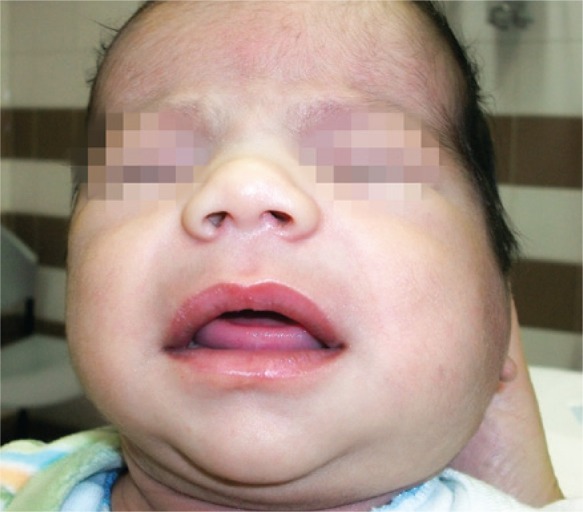
Swelling and left preauricular flushing

**Figure 2:**
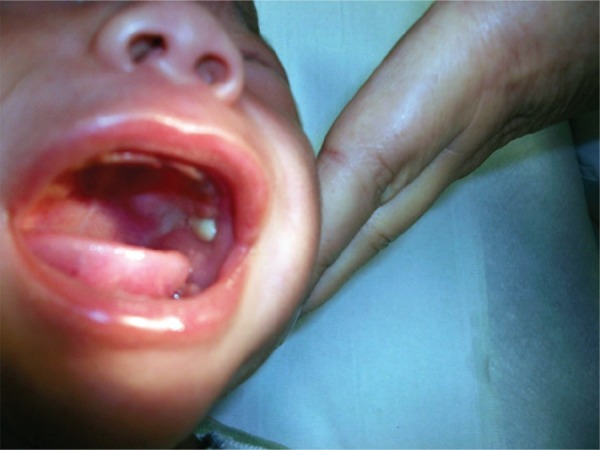
Purulent drainage from Stensen’s duct

The infant was admitted to the urgency service with mild fever, swelling, preauricular flushing and effacement of the mandibular angle with 1 day of evolution (Figure 1). At examination we detected a purulent exudate exiting Stensen’s duct (Figure 2). Laboratory tests revealed: leukocytes 17,000/mm^3^ with 68% neutrophils and C-reactive protein 1.3mg/L. In microbiologic exam of the exudate exiting Stensen’s duct revealed the methicillin-sensitive *Staphylococcus* *aureus*. At hospital admission the newborn began the treatment with intravenous flucloxacillin and gentamicina and after discharge he completed treatment with oral flucloxacilin (after antibiogram). The infant evolved without complications and was discharged in good clinical condition.

This report showed the typical manifestations of a rare disease in newborn, the acute neonatal suppurative parotitis.^(1)-4)^ The etiopathogeny of acute neonatal suppurative parotitis is not completely known and is associated with common predisposing conditions include prematurity, dehydration, and duct stasis,^(1, 2)^ which were not found in our case. This infection often affects male gender and could be bilateral.^(1)-2)^ Purulent drainage from Stensen’s duct is pathognomonic of this condition.^(3)^ The most common etiologic agent found is the *Staphylococcus* *aureus*.^(3)^ The infection has a good prognosis, rare recurrence^(1)-4)^ and complications (facial plasy, fistula, mediastinitis and extension to external auditory canal).
