# Supplementary material for: Facial swelling in newborn
Source: Einstein (Sao Paulo). 2014 Jul-Sep;12(3):384–5. doi: 10.1590/S1679-45082014AI2606 (PMC4872957; doi:10.1590/S1679-45082014AI2606)
Supplement: Translation

# Translation: Tumefacção facial em recém-nascido

**Authors:** Fabiana Fortunato, Irina Carvalheiro, Cristina Novais, Raquel Carreira, Nádia Brito

**Affiliations:** 1 Centro Hospitalar Oeste Norte, Caldas da Rainha, Portugal.

Recém-nascido de 22 dias, do gênero masculino, primeiro filho de pais não consanguíneos, fruto de uma gravidez vigiada de evolução normal, com avaliação analítica e ecográfica sem alterações, parto na 39ª semana, de cesariana, por não progressão do trabalho de parto, com índice de Apgar ao 1º minuto de 9 e, ao 5º minuto, de 10, e peso de nascimento de 3.240g, sem intercorrências no período neonatal. Alimentação com aleitamento materno exclusivo, com adequada progressão ponderal (com 22 dias de vida, tinha peso de 3.650g).

Observado no Serviço de Urgência por febre baixa, tumefacção e rubor pré-auricular esquerdo, com apagamento do ângulo da mandíbula com 1 dia de evolução (Figura 1). À observação, foi detectada adicionalmente saída de exsudado purulento do canal de Stensen (Figura 2). Analiticamente, apresentava leucócitos 17.000/mm^3^ com 68% neutrófilos e proteína C-reactiva de 1,3mg/dL. No exame microbiológico do exsudado do canal de Stensen, foi isolado *Staphylococcus* *aureus *meticilino-sensível. Durante a internação, foi efetuada terapêutica endovenosa, com flucloxacilina e gentamicina, tendo completado tratamento em ambulatório, com flucloxacilina oral (após antibiograma). A evolução decorreu sem complicações, tendo alta clinicamente bem.

**Figura 1:** Tumefacção e rubor pré-auricular esquerda

**Figura 2:** Drenagem de conteúdo purulento no canal de Stensen

O caso descrito demonstra as manifestações típicas de uma doença rara no recém-nascido: a parotidite aguda supurativa neonatal.^(1)-4)^ Sua etiopatogenia não está completamente esclarecida, sendo descritas, como fatores de risco, a prematuridade, a desidratação e a estase ductal,^(1, 2)^ ausentes neste caso. É mais frequente no gênero masculino e pode ser bilateral.^(1, 2)^ O diagnóstico é clínico e seu aparecimento é mais frequente entre o 7º e 14º dia de vida.^(1)-3)^ A drenagem de conteúdo purulento do canal de Stensen é patognomônica.^(3)^ O agente etiológico mais frequente é o *Staphylococcus aureus*.^(3)^ Tem bom prognóstico,^(1)-4)^ sendo raras as complicações (paralisia facial, fístula, mediastinite e extensão ao canal auditivo externo). A recorrência é rara.^(1)-4)^
